# Measuring nurses’ on-shift physical activity and sedentary time by accelerometry or heart rate monitoring: a descriptive case study illustrating the importance of context

**DOI:** 10.1186/s44167-023-00036-2

**Published:** 2023-12-03

**Authors:** Stephanie E. Chappel, Brad Aisbett, Julie Considine, Nicola D. Ridgers

**Affiliations:** 1 Appleton Institute, Central Queensland University, Adelaide, Australia; 2 Institute for Physical Activity and Nutrition (IPAN), School of Exercise and Nutrition Sciences, Deakin University, Geelong, Australia; 3 School of Nursing and Midwifery and Centre for Quality Patient Safety in the Institute of Health Transformation, Deakin University, Geelong, Australia; 4 Centre for Quality and Patient Safety Research, Eastern Health Partnership, Box Hill, VIC 3128 Australia; 5 Alliance for Research in Exercise, Nutrition and Activity, Allied Health and Human Performance, University of South Australia, Adelaide, SA Australia

**Keywords:** Medical-surgical nursing, Nursing, Physical activity, Physical activity monitoring, Sedentary behaviour, Shiftwork

## Abstract

**Background:**

There is debate whether nurses are active enough stemming from differences in measurement tools, clinical contexts, and nursing tasks. A descriptive case study concerning the use of device-based measures in combination with direct observation is presented to examine the effect of the nursing context and the discrepancies between different measurement tools for identifying nurses’ on-shift activity levels.

**Methods:**

Data were collected across seven shifts in medical and surgical wards. Nurses’ activity was assessed using accelerometry and heart rate monitoring, in addition to direct observation. Data graphs were plotted for each shift and measurement device, with direct observations used to contextualise the data and identify discrepancies.

**Results:**

Higher activity levels were recorded on-shift through heart rate monitoring (87%) compared to accelerometry (27%). This pattern was also observed specifically on early, late, and medical ward shifts. Data discrepancies between the two devices stemmed from the shift and (or) ward type, highlighting the importance of understanding the context of nursing duties when assessing nurses’ activity levels.

**Conclusions:**

It is also vital that researchers, policymakers, and practitioners consider how they will measure nurses’ occupational physical activity, which consequently will influence outcomes, and therefore, decisions around the need (or not) for intervention.

**Supplementary Information:**

The online version contains supplementary material available at 10.1186/s44167-023-00036-2.

## Background

Nurses are the largest healthcare workforces globally [1]. They are responsible for the coordination and delivery of healthcare 24 h a day and play a key role in maintaining patient safety [2, 3]. To ensure patient safety and around the clock care, nurses work long shifts with irregular rostering patterns, and undertake physically demanding tasks [4].

In recent years there has been research examining the physical activity levels of nurses on-shift, though the evidence is mixed. Research from a number of countries has shown that nurses either exceed current physical activity recommendations of at least 150 min of moderate- to vigorous-intensity physical activity in a week [4–11] or are highly inactive (not meeting recommended guidelines), thus requiring intervention [12–17]. The investigation of nurses’ physical activity levels on-shift is important as it is known that occupational physical activity levels can have detrimental impacts on an individuals’ health, such as increasing cardiovascular disease risks and mortality [18]. However, one explanation for this paradox is that the balance between occupational physical activity and recovery time is insufficient to achieve health benefits [18]. Furthermore, interventions to improve nurses’ health and wellbeing are being called for and designed based on such mixed findings. As such, before we can determine the need for these interventions to change nurses’ physical activity levels and improve health, we need to accurately capture them.

Nursing is a physically demanding occupation, where the characteristics of nurses’ work and associated levels of physical activity can be variable. For example, during a shift nurses perform both physically demanding tasks (e.g., cardiopulmonary resuscitation, proving care for highly dependent patients with impaired mobility) and accrue up to four hours of sedentary time (SED; [19–22]) during tasks such as documentation, medication preparation, and patient and carer counselling. Further, the work context also influences nursing practice and associated levels of physical activity (e.g., emergency nursing vs. medical or surgical nursing vs perioperative nursing). A major reason for variability in the research to date on nurses’ physical activity is the way in which nurses’ physical activity is operationalised and measured, including whether reported data incorporates both on- and off-shift physical activity (or only one), and what measurement device or approach is used. This highlights the highly context-specific nature of nursing work and need to assess both the domain (e.g., occupation vs. leisure time) and within-shift tasks and duties to characterise nurses’ physical activity.

At work, nurses perform a range of duties and activities, all of which will influence their SED and physical activity levels. For example, when performing cardiopulmonary resuscitation, an essential physically demanding task, nurses performing chest compressions are rotated every two-minutes to manage fatigue and maintain best practice [23]. During two-minute rest periods, nurses may sit (i.e., SED) or stand (i.e., light-intensity physical activity [LPA]) to continue to work as part of the team. In another context, after completing a physically demanding task such as assisting a patient in and out of bed and to shower, another essential physically demanding task, nurses may sit to record patient assessment data and health status. In these examples, the physical activity levels of these tasks are essential for nurses to complete their work and provide a high level of care to their patients, and the SED completed after the task may be essential for recovery [14, 24]. However, SED may also be a necessary part of an essential task. For example, when communicating delicate information to a patient and their family, sitting down fosters a safe, understanding, and comfortable environment compared to potential power imbalances and a sense of distance if standing. These different contexts highlight that both physical activity and SED are necessary and a fundamental part of nursing work [21]. In addition to the context and physicality of nursing tasks, it is important that we also investigate how they are accumulated across a shift and the temporal patterning to provide more insights into the demands of nursing across a shift [25]. For example, the majority of nursing tasks are short in duration (30–60 s; [26]), but if they are performed frequently across a shift or in succession, they can increase the overall physical demand or sedentary nature of the shift. This is in line with previous research suggesting that nursing work is likely to be intermittent due to the nature of the duties, periods of low-intensity activity interspersed by periods of high-intensity activity which is dictated by job and patient demands [4].

Attempting to capture nurses’ SED and physical activity during shifts while considering different tasks and contexts is complicated. Device-based tools such as accelerometers [9–11, 14, 21, 22, 27] and heart rate monitors [28–31] are commonly used in nursing studies. However, inherent device limitations may misclassify nurses’ activity levels on-shift. Previously, the approach used to analyse accelerometer data was limited in its ability to distinguish between sit/stand due to their measurement of acceleration and not postural position (missing standing still) and their location on the body (e.g., hip located devices; [32]). On the other hand, heart rate monitoring can be influenced by external factors (i.e., stress, which could result in high heart rates for tasks with little to no movement), has weak relationships with lower intensities, and has time lags to intensity changes [33]. Despite advances in the assessment of behaviours using such devices (e.g., machine learning [34, 35], neither approach provides insights in nursing tasks undertaken during a shift. The inaccuracies in current measurement practices can have implications for researchers, policymakers, and practitioners using such approaches to determine whether interventions for nurses’ activity levels at work are required. To accurately describe nurses’ physical activity and SED, multiple measurement approaches may be required to overcome respective limitations associated with specific device-based measures. For example, using both accelerometry and heart rate monitoring can address the limitations of each other, and devices have been developed for this specific reason. However, this still leaves a gap in being able to accurately identify which device is capturing the correct intensity information at particular times and for specific nursing tasks. To help identify discrepancies between the devices an additional measurement approach, such as direct observation, could be used to allow time-stamped information on the exact task and movements being completed. Therefore, the aim of this article was to present a descriptive case study of the use of device-based measures overlayed with direct observation data to examine the importance of considering the nursing setting and context. Specifically, the identification of nurses’ on-shift SED and physical activity levels were explored using different measurement tools.

## Methods

A convenience sample of nurses were recruited from one medical and one surgical ward from an acute care hospital in Melbourne, Australia. All nurses were sent information about the study via email. Four female nurses, two from each ward (1 enrolled nurse and 3 registered nurses), provided written informed consent to participate. In total, seven shifts resulting in 54 h of data and 38,880 data points per measurement tool were collected. Ethical approval was obtained from Eastern Health (E12-2016) and Deakin University (2017-038) for the study. This study is reported in line with the STROBE guidelines (Additional file 1).

Data were collected across two consecutive shifts, except for one surgical nurse that did only one shift. Two nurses worked a late shift (1400–2200) followed by an early shift (0700–1500). The remaining two nurses worked an early shift followed by a late shift, and one early shift, respectively. Before starting their shift, the nurses’ age, and height (tape measure) and weight (weighing chair) were obtained. Nurses were fitted with two monitors to be worn during their shift: an ActiGraph accelerometer and a Polar heart rate monitor. During each shift, investigators took observational notes of the entire shift. Specific nursing tasks were only filmed when care was delivered with the bed curtains open (in public view) and with consent from all present (e.g., patients and other nursing staff). Monitoring devices were removed at the end of each shift. Observations occurred until the end of the early shifts and at 2000 for late shifts for investigator safety.

## Materials

### ActiGraph activity monitor

Each nurse was fitted with a GT3X + ActiGraph activity monitor (Pensacola, FL, USA). This is a small light-weight device (46 × 33 × 15 mm) worn on the right hip via an elastic strap (see Additional file 2: Fig. S1). The hip location was to align with nursing guidelines of nothing worn on the wrists. Raw acceleration data were sampled at 30 Hz. Data were downloaded in 5-s epochs and processed using a customised Excel macro. Validated cut-points determined the time spent in LPA (1.50–2.99 metabolic equivalents [METs]), moderate-intensity physical activity (MPA; 3.00–5.99 METs) and vigorous-intensity physical activity (VPA; ≥ 6.00 METs [36]). SED was defined as ≤ 100 counts per minute [37]. Non-wear time was set at 90 min of consecutive zeros to account for longer periods of no movement (i.e., breaks). As the nurses only wore the monitor on-shift the wear time could be verified by the research team.

### Polar heart rate monitor

Nurses were fitted with a Polar heart rate monitor (Polar, Electro Oy, Kempele, Finland) via an elastic strap at the base of the sternum (see Additional file 3: Fig. S2). The monitor collected data in 5- intervals. The maximal heart rate of each nurse was calculated using the Equation (38):

$${\text{Maximal Heart Rate }} = \, 0.{7} \times \left( {{2}0{7 }{-}{\text{ age}}} \right)$$

The calculated maximal heart rate was used to determine each nurses’ relative heart rate intensity into SED, LPA, MPA, and VPA using pre-determined thresholds (see Additional file 4: Table S1 [39]).

### Direct observation

Across a shift, nurses were directly observed by at least two members of the research team. One researcher recorded observational notes that documented the task the nurse was completing, the movements involved, and the time the task stated and ended. The second researcher used a JVC video camera (GZ-R10R—Quad Proof Everio HD Camcorder) to record specific nursing tasks. Where possible, only the nurse was filmed, and patients were informed that filming was occurring and could decline the filming of their care/treatment. ‘Closed curtain’ tasks were not filmed, but described by the nurse once they came back out from behind the curtain. Given the range of tasks nurses undertake during a shift, the observations were coded as direct patient care (e.g., transferring a patient from a bed to a chair and showering a patient), indirect patient care (e.g., medication preparation and paperwork), and break times (e.g., tea and lunch/dinner [29]). This method was chosen to allow identification and discussion of discrepancies between measurement devices given that not all nurses completed the same tasks during their shift. Furthermore, this has been used in previous studies [29]. The frequencies of each of the tasks across the shifts were calculated.

### Data analysis

The proportion of time that nurses spent SED, and in LPA, MPA and VPA during shifts was obtained for both heart rate and accelerometry data. These calculations were determined by dividing the total duration of the shift by the time spent in SED, LPA, MPA, and VPA. For the purpose of this case study, absolute minute level data is not provided as the main focus of the study is on identifying the discrepancies between measurement devices using direct observation, and the durations of the shifts varied. Means and standard deviations (SD) were calculated for all data and summarised according to shift (all, early, late) and ward type (medical, surgical). Where required, IBM SPSS Statistics for Windows (Version 26) was used for analysis. Heart rate and accelerometer data were matched by day and time and plotted for each nurse and shift. Where differences between the two devices were noted, direct observation information was used to contextualise the task. In addition, the 2011 Compendium of Physical Activities were used as a reference point to identify the typical MET classifications of activities/tasks to match each device’s outcomes against [40]. Case studies are presented to highlight how different tasks and task-timing across shifts may explain conflicting results obtained from monitoring devices.

## Results

Data were collected from four early shifts and three late shifts. The nurses were, on average, 36 years old, 165 cm tall and weighed 68 kg. Data collected from each shift ranged from: 6.75 h (early shift) to 7.33 h (late shift). During a shift, nurses had two breaks, a tea break (average 27 min) and a lunch/dinner break (average 37 min). The most frequently performed tasks across all shifts were those involving direct patient care (Table 1).

**Table 1:** Frequency of nursing tasks across each shift

Tasks	Nurse 1 late shift	Nurse 1 early shift	Nurse 2 early shift	Nurse 3 late shift	Nurse 3 early shift	Nurse 4 early shift	Nurse 4 late shift	Total
**DIRECT PATIENT CARE**								
General observations (including bloods, blood pressure, blood sugar checks)	5	6	3	6	15	9	9	**53**
Showering patient						3		**3**
Dressing/Changing patient		1	1			2		**4**
Feeding patient					2	2		**4**
Giving medication	1		2	3	6	7	3	**22**
Assisting patient to walk/move	1	6		2	3	5	2	**19**
Moving patient in bed	2				2	3	5	**12**
Transferring patient between beds, chair, trolley etc	1	2	5	3		5	4	**20**
Wash patient in bed		1				1		**2**
Dressing wounds	1	2	1	3			2	**9**
Bed pan change	2			2		1	1	**6**
Apply stockings			1					**1**
MET call					1		2	**3**
**Total**	**13**	**18**	**13**	**19**	**29**	**38**	**28**	**158**
**INDIRECT PATIENT CARE**								
Medication preparation	3	3	2	5	9	6		**28**
Moving equipment	1	2	3	1	5	9	3	**24**
IV set up	1	1	2	3	1	2	4	**14**
Cleaning	1		2	1	1	3	2	**10**
Paperwork	2		3	2	8	4		**19**
Make bed		2	1		1	4	1	**9**
Walking	2	3			1	2	1	**9**
Talking with patient					4			**4**
**Total**	**10**	**11**	**13**	**12**	**30**	**30**	**11**	**117**

*IV* intravenous, *MET* emergency medicine call

### On-shift activity levels

The average proportion of time spent in different activity intensities according to accelerometry and heart rate data are presented in Additional file 4: Table S2. According to accelerometry data, nurses spent the majority of a shift SED (73%), with limited time engaging in high-intensity activity (~ 5%). However, according to heart rate data, nurses spent the majority of a shift engaging in high-intensity physical activity (~ 52%), with limited time spent SED (13%). This pattern of accelerometry data finding SED to be the highest proportion of a shift and high-intensity activity the smallest and vice versa for the heart rate data was consistent across early, late, and medical wards shifts. Interestingly on surgical ward shifts, the highest proportion of time according to heart rate data was attributed to LPA.

### Case studies

**Fig. 1:**
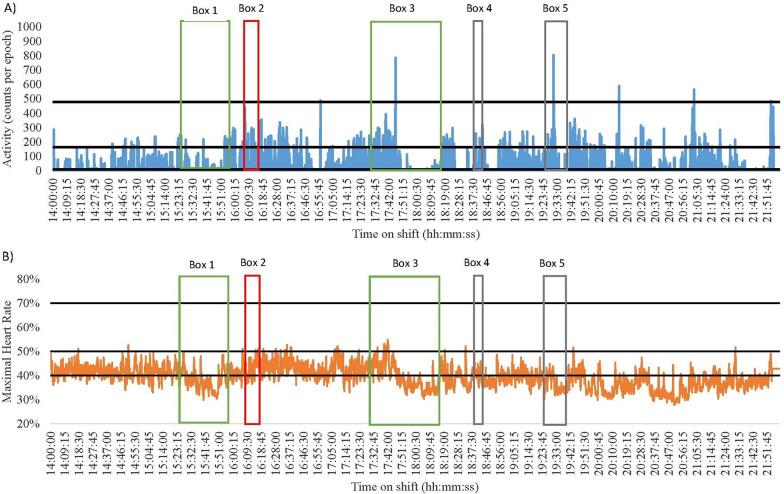
Nurse 1 Late Shift on a Medical Ward. Green = break; Red = direct patient care tasks; Grey = indirect patient care tasks. **A** Data captured with accelerometry. Black lines indicate an accelerometry cut-points: Sedentary (SED) is < 8 counts per epoch; Light-intensity physical activity (LPA) is 8–163; Moderate-intensity physical activity (MPA) is 164–477; Vigorous-intensity physical activity (VPA) is > 478. **B** Data captured for heart rate monitoring. Black lines indicate a heart rate cut-points: SED is < 40%; LPA is 41–50%; MPA is 5–70%; VPA is > 71%

**Fig. 2:**
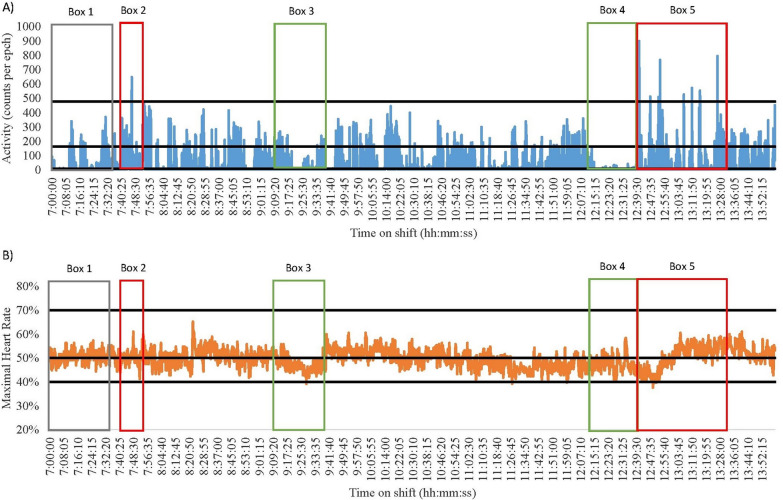
Nurse 1 Early Shift on a Medical Ward. Green = break; Red = direct patient care tasks; Grey = indirect patient care tasks. **A** Data captured with accelerometry. Black lines indicate an accelerometry cut-points: Sedentary (SED) is < 8 counts per epoch; Light-intensity physical activity (LPA) is 8–163; Moderate-intensity physical activity (MPA) is 164–477; Vigorous-intensity physical activity (VPA) is > 478. **B** Data captured for heart rate monitoring. Black lines indicate a heart rate cut-points: SED is < 40%; LPA is 41–50%; MPA is 5–70%; VPA is > 71%

**Fig. 3:**
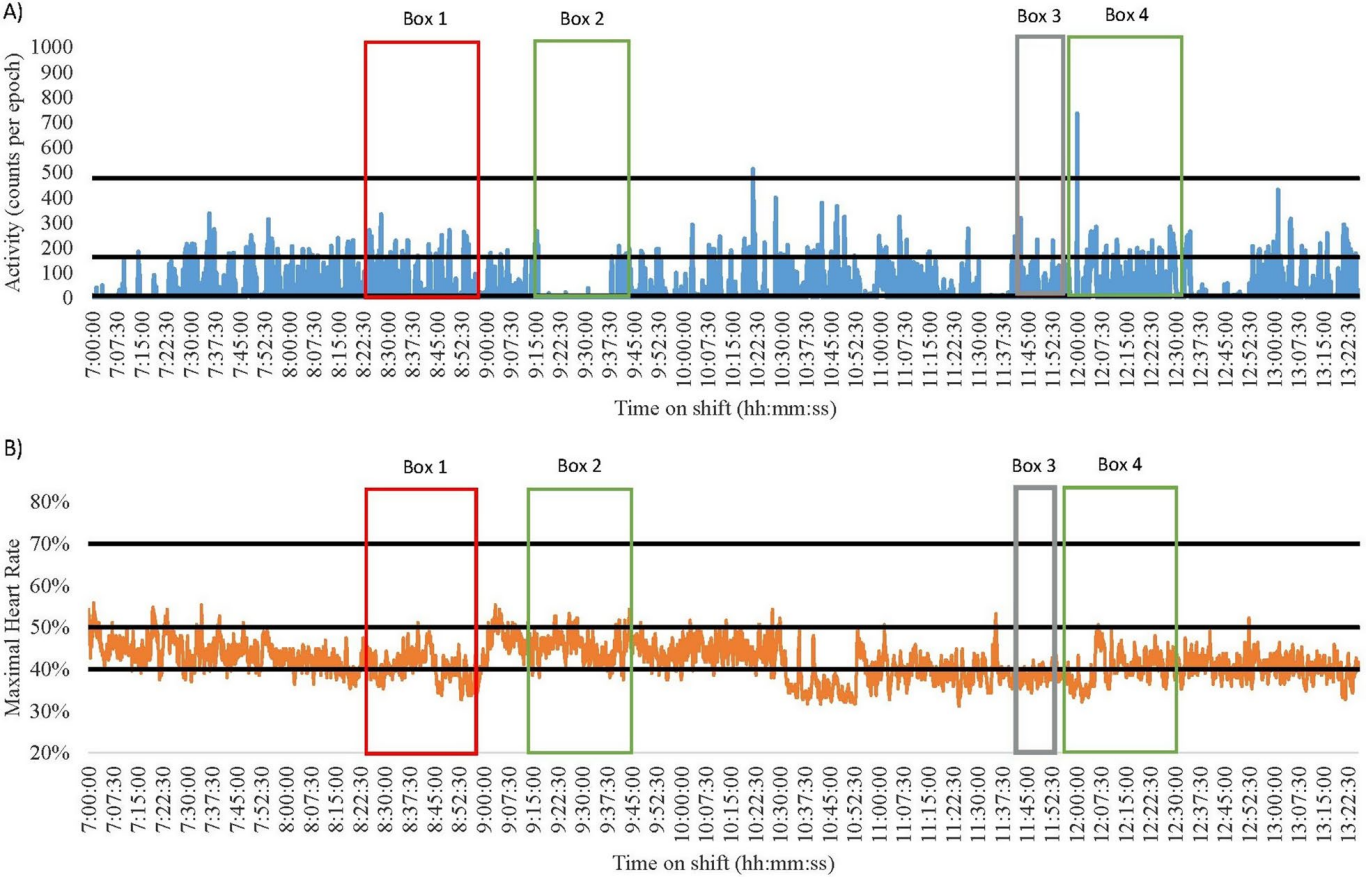
Nurse 2 Early Shift on a Surgical Ward. Green = break; Red = direct patient care tasks; Grey = indirect patient care tasks. **A** Data captured with accelerometry. Black lines indicate an accelerometry cut-points: Sedentary (SED) is < 8 counts per epoch; Light-intensity physical activity (LPA) is 8–163; Moderate-intensity physical activity (MPA) is 164–477; Vigorous-intensity physical activity (VPA) is > 478. **B** Data captured for heart rate monitoring. Black lines indicate a heart rate cut-points: SED is < 40%; LPA is 41–50%; MPA is 5–70%; VPA is > 71%

**Fig. 4:**
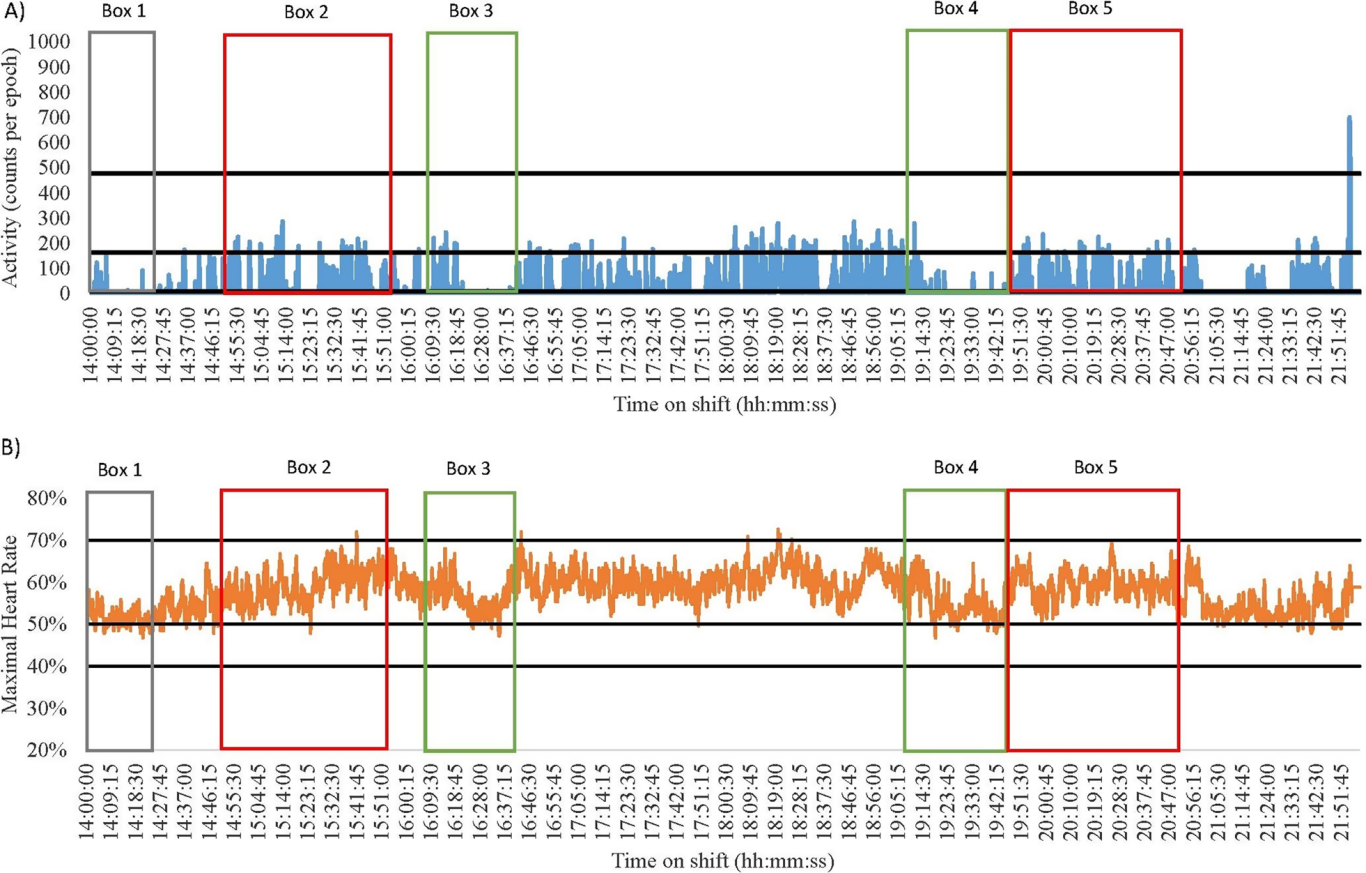
Nurse 3 Late Shift on a Medical Ward. Green = break; Red = direct patient care tasks; Grey = indirect patient care tasks. **A** Data captured with accelerometry. Black lines indicate an accelerometry cut-points: Sedentary (SED) is < 8 counts per epoch; Light-intensity physical activity (LPA) is 8–163; Moderate-intensity physical activity (MPA) is 164–477; Vigorous-intensity physical activity (VPA) is > 478. **B** Data captured for heart rate monitoring. Black lines indicate a heart rate cut-points: SED is < 40%; LPA is 41–50%; MPA is 5–70%; VPA is > 71%

**Fig. 5:**
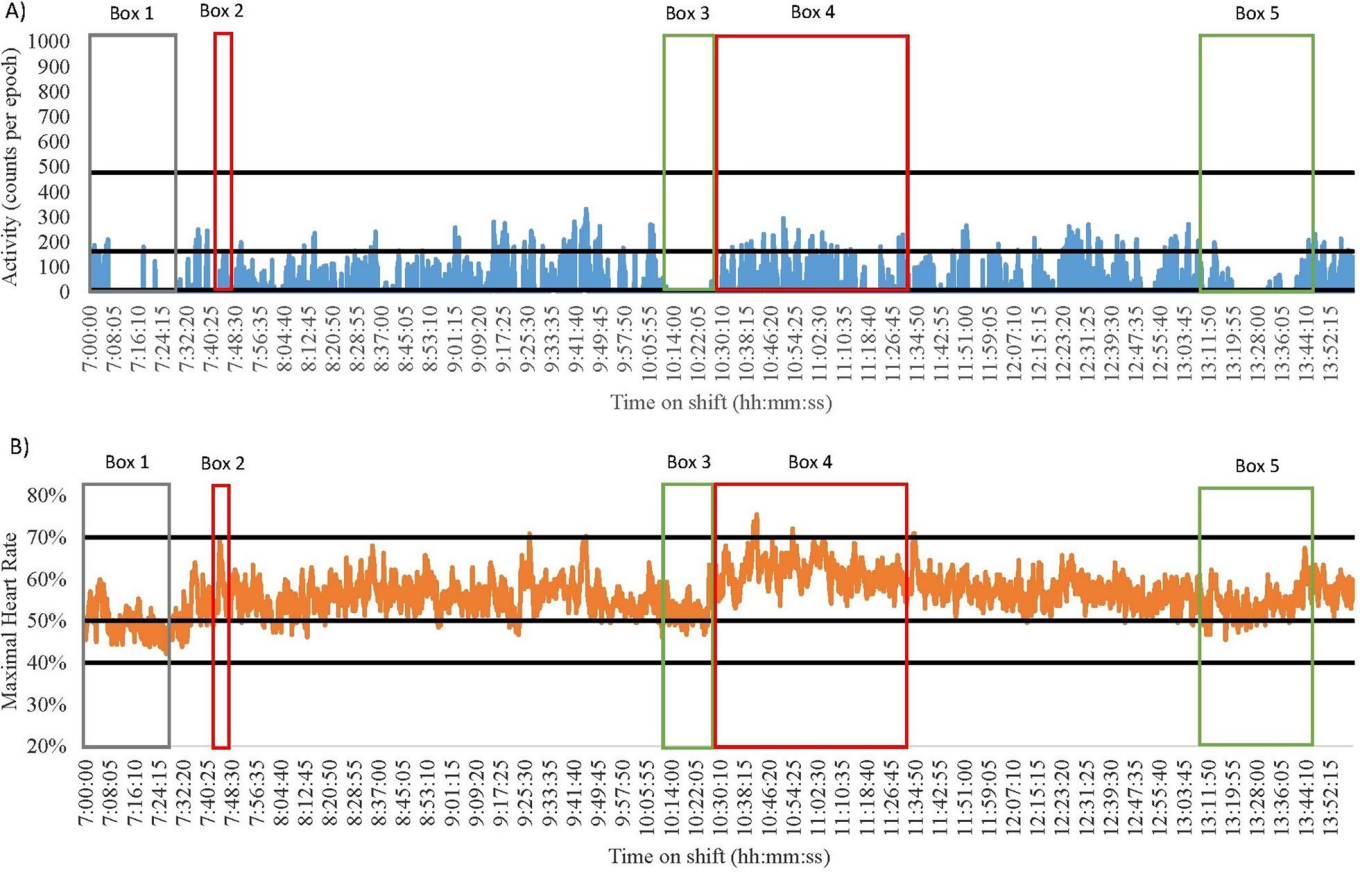
Nurse 3 Early Shift on a Medical Ward. Green = break; Red = direct patient care tasks; Grey = indirect patient care tasks. **A** Data captured with accelerometry. Black lines indicate an accelerometry cut-points: Sedentary (SED) is < 8 counts per epoch; Light-intensity physical activity (LPA) is 8–163; Moderate-intensity physical activity (MPA) is 164–477; Vigorous-intensity physical activity (VPA) is > 478. **B** Data captured for heart rate monitoring. Black lines indicate a heart rate cut-points: SED is < 40%; LPA is 41–50%; MPA is 5–70%; VPA is > 71%

**Fig. 6:**
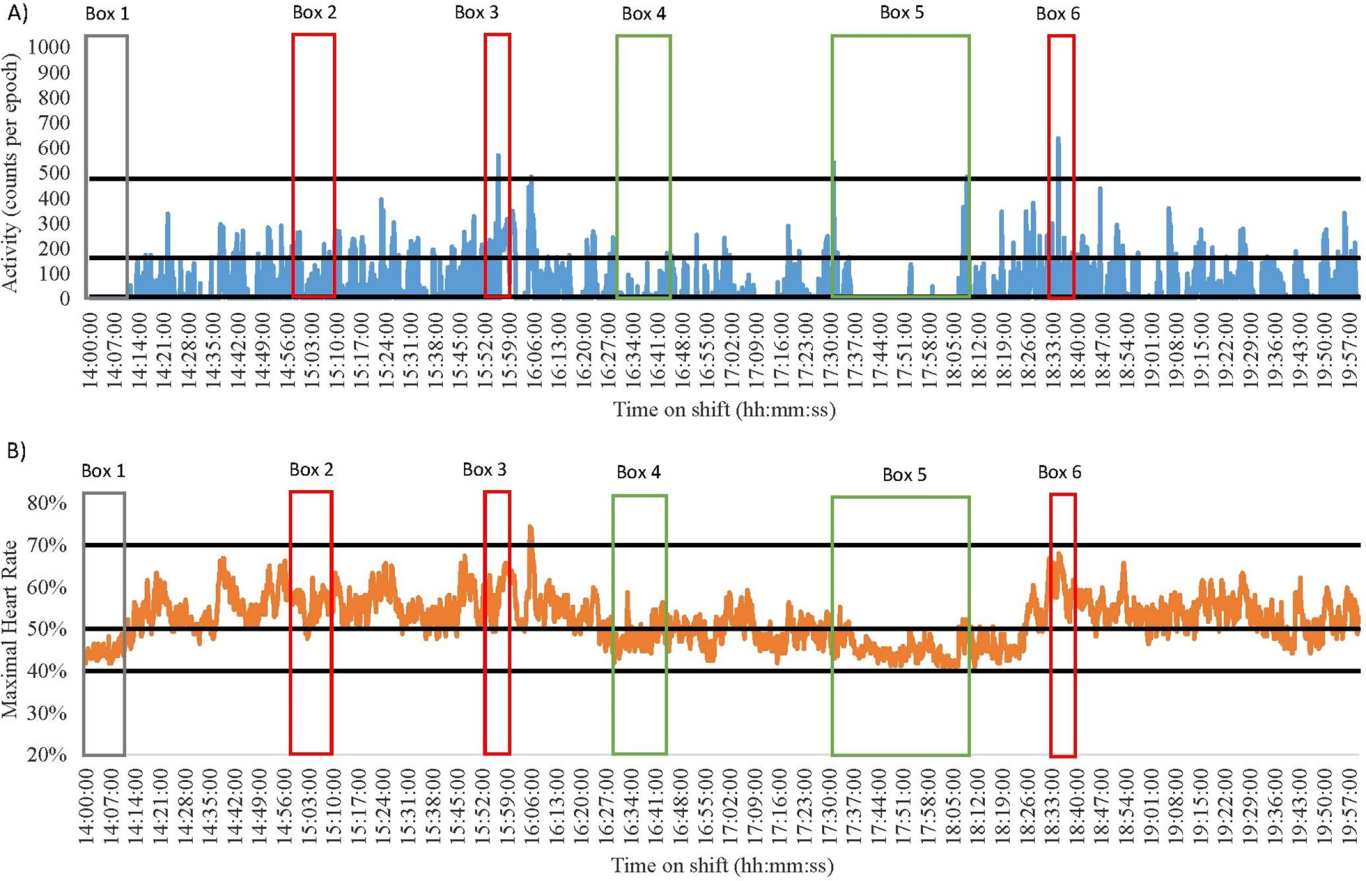
Nurse 4 Late Shift on a Surgical Ward. Green = break; Red = direct patient care tasks; Grey = indirect patient care tasks. **A** Data captured with accelerometry. Black lines indicate an accelerometry cut-points: Sedentary (SED) is < 8 counts per epoch; Light-intensity physical activity (LPA) is 8–163; Moderate-intensity physical activity (MPA) is 164–477; Vigorous-intensity physical activity (VPA) is > 478. **B** Data captured for heart rate monitoring. Black lines indicate a heart rate cut-points: SED is < 40%; LPA is 41–50%; MPA is 5–70%; VPA is > 71%

**Fig. 7:**
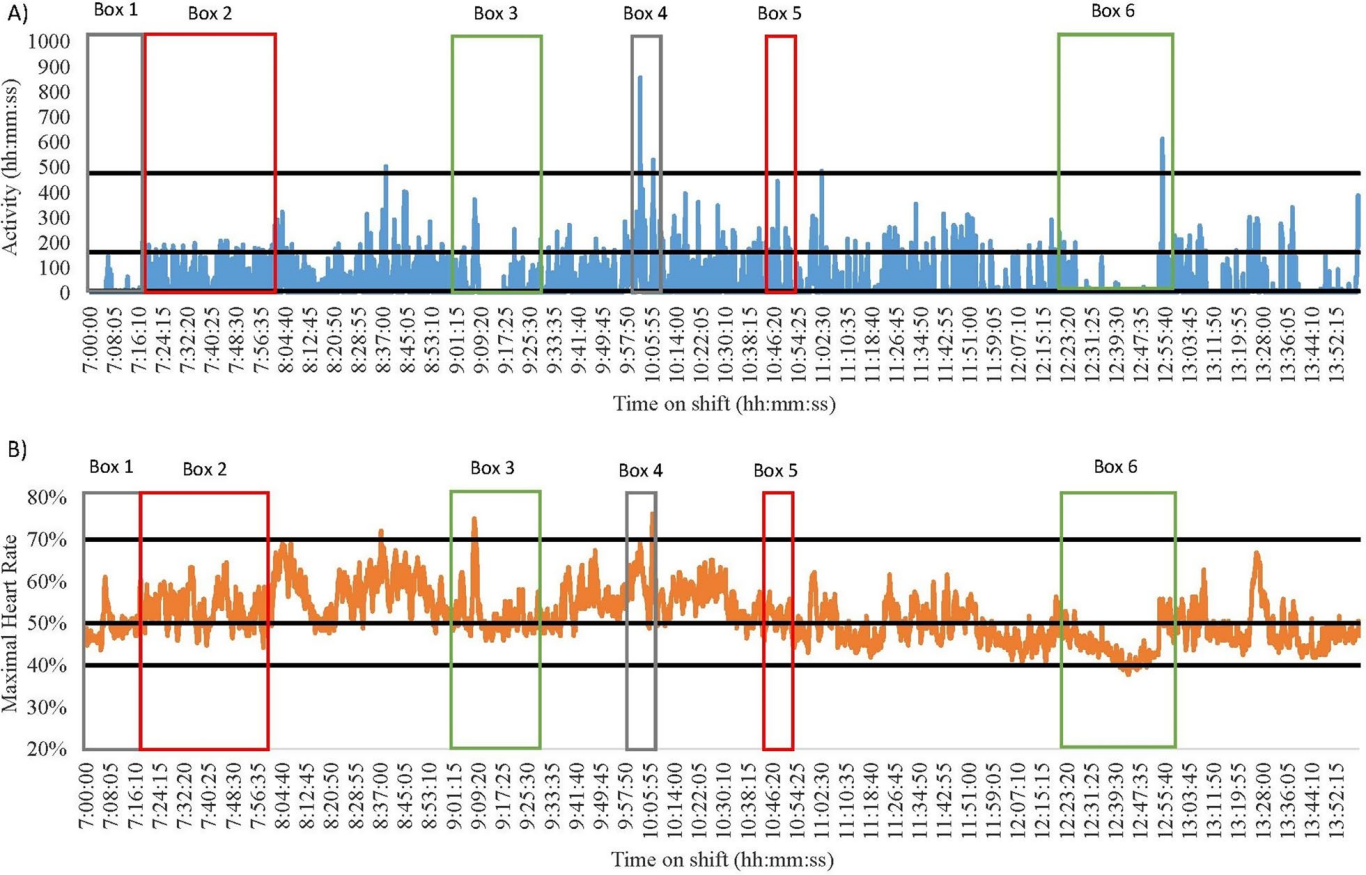
Nurse 4 Early Shift on a Surgical Ward. Green = break; Red = direct patient care tasks; Grey = indirect patient care tasks. **A** Data captured with accelerometry. Black lines indicate an accelerometry cut-points: Sedentary (SED) is < 8 counts per epoch; Light-intensity physical activity (LPA) is 8–163; Moderate-intensity physical activity (MPA) is 164–477; Vigorous-intensity physical activity (VPA) is > 478. **B** Data captured for heart rate monitoring. Black lines indicate a heart rate cut-points: SED is < 40%; LPA is 41–50%; MPA is 5–70%; VPA is > 71%

Comparisons between accelerometer and heart rate data by shift and ward types highlighted different patterns in activity levels. As such, descriptive case studies have been developed to discuss and understand the variability observed (Figs. 1, 2, 3, 4, 5, 6 and 7). Each of these figures represents one nurses shift and presents for the entire shift the (A) accelerometry data in blue and (B) heart rate data in orange. The black lines on the graphs represent the different cut-points used to identify SED, LPA, MPA and VPA. The coloured boxes represent points of interest regarding discrepancies between the two measurement devices; green is a break, red is direct patient care tasks, and grey is indirect patient care tasks. While these case studies are context-specific and therefore not broadly generalisable, these provide insights into issues concerning the measurement of nurses’ physical activity levels at work. Table 2 provides the typical sequence of duties performed on early and late shifts for information.

**Table 2:** Structure of typical shifts

Typical activity			
Time	Morning shift	Time	Afternoon shift
0700	Start of shift Indirect patient care Direct patient care	1400	Start of shift Indirect patient care Direct patient care
0800	Direct patient care	1500	Afternoon break
0900	Direct patient care Morning break	1600	
1000		1700	Indirect patient care Dinner break
1100		1800	
1200	Indirect patient care Lunch break	1900	
1300	Indirect patient care	2000	Direct patient care
1400	Indirect patient care	2100	Indirect patient care
1500	Finish shift (1500–1530)	2200	Finish shift (2130–2230)

This is an outline of the typical structure of a shift; however, nurses’ activities are patient dependent and may occur at different times. Direct patient care includes transferring patients from bed to chair, bed to trolley or chair to bed, assisting patients to walk, showering patients, administration of medications, and implementation of therapies. Indirect patient care includes medication preparation, paperwork and walking between rooms and wards. The gaps across the shift represent particular times where nurses’ work is not structured, and their activity solely will depend on patient care needs

#### High SED vs. low SED shifts

Data from two nurses demonstrate how no two shifts are alike. Nurse 1 from a medical ward had a highly SED late shift (55% and 71% according to heart rate and accelerometry, respectively, Fig. 1). In contrast, Nurse 4 from a surgical ward had a low SED shift (0% and 59% as measured by heart rate and accelerometry, respectively, Fig. 6). The differences in SED time may be explained by the different types of tasks completed (direct vs. indirect vs. combination), wards worked on, measurement device used, or an interaction between any of these. Nurse 1 frequently performed direct and indirect tasks, compared to Nurse 4 who more frequently engaged in direct patient care tasks (See Table 1). The higher engagement in indirect nursing tasks by Nurse 1 may explain the large differences in SED across the shifts. For example, Nurse 1 spent more time completing paperwork whilst sitting (see Fig. 1 Box 4). On the other hand, Nurse 4 engaged in more direct patient care tasks such as changing a catheter bag and completing patient transfers (see Fig. 6 Box 2 and 6). This may further be explained by the ward the nurse was working on, with surgical patients sometimes requiring more assistance than medical patients, resulting in Nurse 4 have lower SED time. Finally, there may also be some misclassification of certain activities by the measurement tools. For example, in Fig. 6 Box 2 Nurse 4 was engaging in direct patient care tasks (typically 3 METs [40]) that accelerometry measured as SED-LPA, but heart rate monitoring was closer in its measurement suggesting the activity was MPA.

#### High physically demanding and SED shifts

Several shifts were both highly physically demanding and highly SED according to the devices. Of note were the late shift of Nurse 1 (medical) and the early shift of Nurse 2 (surgical). Nurse 1 spent 55% and 71% of their shift SED, and 45% and 29% of the shift engaged in physical activity (heart rate and accelerometry, respectively, Fig. 1). Nurse 2 was SED for 33% and 75% of the shift and engaged in physical activity for 67% and 25% of their shift (heart rate and accelerometry, respectively, Fig. 3). The majority of both nurses’ SED was accumulated whilst sitting down during tea (see Fig. 1 Box 1 and Fig. 3 Box 2) and lunch breaks (see Fig. 1 Box 3 and Fig. 3 Box 4). Furthermore, both nurses frequently engaged in indirect nursing tasks that typically required little or no movement, such as medication preparation and paperwork (See Table 1). Although MPA levels were similar between the two nurses very different tasks were completed. Nurse 2 engaged in indirect patient care tasks that involved moving and using heavy equipment (see Fig. 3 Box 3). Conversely, Nurse 1 spent more time assisting bariatric patients to move (see Fig. 1 Box 2).

#### Accelerometry and heart rate differences

When overlaying data collected using accelerometers and heart rate monitors with directly observed tasks, discrepancies were noted between the energy cost of the task and the energy cost recorded using the devices. For example, the early and late shifts of Nurse 3 (medical) and 4 (surgical) had noticeable differences in the time spent SED and in MPA (See Figs. 4, 5, 6 and 7). During breaks when nurses were observed mainly sitting (typically 1.5 METs [40]), accelerometry classified nurses as engaging in little movement (below the SED threshold). However, heart rate remained elevated (38–75% maximal heart rate) misclassifying activity levels as above the LPA threshold (see the differences between A and B in Fig. 4 Boxes 3 and 4; Fig. 5 Boxes 3 and 5; Fig. 6 Boxes 4 and 5; Fig. 7 Boxes 3 and 6). At the start of shifts both nurses engaged in indirect patient care tasks, such as patient handover (typically 2–3 METs [40]); see Figs. 4, 5, 6 and 7 Box 1). These tasks were identified by heart rate monitoring as yielding LPA-MPA (42–56% maximal heart rate), with observations confirming nurses were standing and walking to complete tasks. However, accelerometry did not record any LPA during the handover period even though nurses were observed standing. This is most likely due to the lack of acceleration occurring as the nurses were most likely standing still [32]. Several direct patient care tasks completed by Nurse 3, including patient transfers (typically 4 METs [40]) and assisting patients to walk (typically 3.5 METs [40]; see Fig. 4 Box 5 and Fig. 5 Box 2), which also resulted in a higher activity classification from heart rate data (45–73% maximal heart rate) than from accelerometery data. Conversely, both Nurse 4’s shifts had moments where accelerometry counts were higher (i.e., greater movement) than the relative activity intensity as indicated by heart rate. This was found during both direct patient care tasks (e.g., preparing an intravenous drip; typically 3 METs [40]; see Fig. 6 Box 3), transferring patients (typically 4 METs [40]; see Fig. 6 Box 6), and taking blood pressure (typically 3 METs [40]; see Fig. 7 Box 5), and indirect tasks (e.g., walking off ward to the pharmacy (typically 3.5 METs [40]; see Fig. 7 Box 4).

## Discussion

The aim of this study was to present a descriptive case study of the use of device-based measures overlayed with direct observation data to examine the effect of the nursing context and the discrepancies between different measurement tools for identifying nurses’ on-shift activity levels. Based on the accelerometry data, the ratio of SED to physical activity time across all shifts was 73:27, and higher LPA was recorded by surgical nurses. In contrast, heart rate responses returned a 13:87 ratio of SED to physical activity, and nurses working an early shift engaged in more LPA. Interestingly, nurses spent a greater proportion of their shift engaged in MPA according to heart rate, but only small differences were evident for LPA levels between the devices. Given the discrepancies observed by the devices, it was important to contextualise the nurses’ shifts through direct observations. The case studies presented have implications for researchers, policymakers, and practitioners who previously relied on objective measures alone to determine the need (or not) for physical activity interventions for nurses.

Differences were evident in nurses’ on-shift physical activity levels according to the device used. The majority of nursing shifts were spent SED or engaged in LPA when quantified using accelerometry, yet when analysing heart rate data, the majority of a shift was spent engaged in MPA. This may explain, in part, the current conflictions within the literature of nursing being classified as SED or LPA on-shift [4, 9–11]. Other reasons for discrepancies may include the type or sequencing of tasks performed on-shift. For example, according to heart rate monitoring nurses’ break times were classed as MPA, even though they were observed sitting down. This was most likely as a result of completing a physical task prior to their break and the short break durations (~ 30-min), which may result in an elevated heart rate and without enough time to recover. Interestingly, this has been suggested as one of the explanations behind physical activity paradox, where occupational physical activity does not have the same effects as leisure time physical activity [18]. Discrepancies in the data may also be explained by the context of the ward or shift type with unpredictable work driven by patient needs [41]. For example, surgical nurses, according to accelerometry, engaged in more LPA than those on the medical ward. This could be a result of surgical ward nurses spending more time in patient assessment activities (~ 50 min across a 12-h shift; [42]) compared to medical ward nurses (~ 39  across a 12 shift; [43]), that may have often been completed whilst standing. Furthermore, from the current observations the surgical ward nurses engaged in on average 26 occasions of direct care patient tasks per shift compared to medical ward nurses 20 (see Table 1), which was most likely attributed to these patients observed to require more assistance and frequent patient assessments as they were in the immediate post-operative period. Further, according to heart rate data, working on an early shift involved more LPA than working on a late shift. This could be as a result of the early shift requiring nurses to assist with more activities of daily living (e.g., showering, feeding and dressing; typically 2.3–4 METs [40]; Table 1). This is in line with previous literature finding that across a 12-h shift, medical ward nurses spent ~ 44 assisting patients with activities of daily living across a 12-h shift [43] whilst surgical ward nurses spent ~ 23 min [42]. Finally, it should be noted that nurse-patient ratios, staff mix, and the number of patients in a nurse’s direct care within a shift are additional factors that could influence nurses’ accumulation of physical activity and SED time through the required work tasks [44–46]. These results highlight that understanding the specific contexts’ (e.g., nurses’ roles, shift-types, and tasks) in which monitoring takes place are critical considerations prior to considering strategies to increase physical activity levels, for example, which may not necessarily be required.

Understanding the context of nursing work is important, however, researchers also need to consider the limitations of the measurement device used. For example, Nurse 3 was observed changing a dressing on a patient’s face whilst standing (see Fig. 4 Box 2), which is a LPA behaviour according to the associated energy cost (3 METs [40]), but it was misclassified as a MPA by heart rate monitoring. It is likely that the stress of performing a procedure in a delicate location like the face and trying to prevent procedural pain increased stress which therefore elevated heart rate, despite being a low-intensity task. This observed example is just one of the situations in a nursing environment where stress can influence physical activity intensity levels when little to no movement is being completed. Other may include tough conversations with patients and/or family or preparation for a difficult/challenging task (e.g., informing a patient and their family that they are terminal). Accelerometry on the other hand, misclassified transferring a patient (typically 4 METs [40]; Fig. 6 Box 2) as SED, yet the task would typically be described as physically demanding (64.7% of maximal heart rate [29]). This is most likely a result of the lack of acceleration associated with the task as the nurse remained standing still but bending, twisting, or lifting [32]. Across the length of a shift, such differences could have implications for the quantification of physical activity and SED over time [32]. Indeed, in the current study accelerometery resulted in higher SED time (73%), and heart rate monitoring resulted in higher physically active time (87%). Overall, this reinforces the need to determine the types of tasks that nurses on different wards and shifts are performing. Moreover, the need to potentially combine measurements of energy expenditure and heart rate together to examine the energy cost of activities where possible as they will counter one another. In doing so, conclusions on nurses’ occupational physical activity and SED can be more accurate than through device-based measurement alone.

Nursing is a complex occupation, which was clearly demonstrated in this case study. Therefore, careful consideration is required when deciding how to measure nurses’ physical activity. In combination, these devices provide a more detailed picture of the mechanical (accelerometer) and physiological (heart rate) strain. However, alone either device may misrepresent the duration and intensity of specific nursing tasks [47]. Importantly, collecting additional information about the shift context and types of tasks provides more detailed insights on nurses’ physical activity and may explain discrepancies. Direct observation was used as it provides rich information about shift activity, but it is costly over sustained periods and with larger samples [48]. Combining device-based assessments with self-reported contextual measures (e.g., diaries and logbooks) could be a useful, less costly approach and has been used within the literature [9–11]. However, further exploration is required to determine the best method of collecting contextual data whilst limiting participant burden given the magnitude of nurses’ tasks and direct patient safety responsibilities. Future researchers should also consider the use of different devices that can complement the data from accelerometer or heart rate monitoring. Inclinometers measure postural positions of the body as sitting/lying, standing, and stepping [49]. Paired with accelerometry or heart rate monitoring, inclinometry may account for some of their limitations. For example, accurately distinguishing standing from sitting, or isolating the intensity and duration of static tasks (e.g., supporting patients in standing postures). Recently, inclinometer and accelerometer data were used in studies of emergency nurses where nursing was classified as LPA (most standing time) or SED, respectively [9–11]. Given that direct observations suggest nursing typically involves more standing time than other postural behaviours, an inclinometer may provide a more accurate representation of activity levels in a nursing environment. Furthermore, as newer analytical methods are developed, including machine learning techniques, it is possible that some of the limitations of these monitoring devices may be addressed.

This descriptive case study had several strengths. This includes the use of two different measurement tools that are regularly used to capture nurses’ SED and physical activity, supplemented by the direct observation. Second, the seven nursing shifts captured resulted in 54 h of data collection and 38,880 data points per measurement tool. Additionally, the combination of high-resolution filming of a shift with heart rate and accelerometry measures produced invaluable, nuanced insights into nurses’ work. However, there are limitations that need to be noted. First, this study was a small case study with four nurses across medical and surgical wards at one hospital. As such, it is not possible to generalise these findings further. Furthermore, whilst this study provides novel important findings, future studies should be done using larger sample sizes (of participants and of shifts) to explore this in further depth, including the validity and reliability of accelerometry and heart rate monitoring in a nursing environment. Second, only the frequency of tasks was captured, limiting in-depth knowledge on the activities completed by nurses. An area for future research should be understanding the duration of specific nursing tasks, where the majority typically last only 30–60 s [26]. Third, information on the nurse-to-patient ratio and number of patients in each nurse’s care at the time of the observation were not recorded. Future work should consider capturing this data to help with contextualising physical activity and SED activities further. Fourth, resting heart rate data were not collected for participants, therefore, only maximal heart rate could be used to obtain activity levels. This method is not as accurate as using heart rate reserve which takes into account an individual’s fitness levels [33]. Finally, as mentioned earlier “closed curtain” tasks were not directly observed but rather described to the researcher by the nurse. Whilst this may have resulted in the “closed curtain” tasks not being accurately captured, it enabled some data to be collected for analysis.

## Conclusions

This case study highlights that the way in which nurses’ on-shift SED and physical activity are measured, and how the context of the work influences conclusions on the physicality of nursing work. The findings demonstrate the need to understand the context of the nursing shift (early or late), the patient population (medical or surgical), and the timing/sequencing of work tasks. Further research using much larger participant numbers is needed to evaluate the influences that different measurement devices may have on quantifying and drawing conclusions on nurses’ activity levels. This future research could confirm observations from the current case study in that multiple measurement techniques may be required to understand the complexity of this occupation and accurately document nurses’ activity levels.

## Supplementary Information

**Additional file 1.** STROBE Statement—checklist of items that should be included in reports of observational studies

**Additional file 2: Figure S1.** ActiGraph accelerometer.

**Additional file 3. Figure S2.** Polar heart rate monitor.

**Additional file 4:** **Table S1. **Physical activity intensities as a percentage of maximal heart rate. **Table S2. **Average percentage of time (%) spent in different activity levels from accelerometry and heart rate data.

## Data Availability

The datasets generated and/or analysed during the current study are not publicly available due to privacy and confidentiality concerns and the ethical approval at the time but are available from the corresponding author on reasonable request.

## Abbreviations

LPA: Light-intensity physical activity
MET: Metabolic equivalents
MPA: Moderate-intensity physical activity
SED: Sedentary time
VPA: Vigorous-intensity physical activity
