# Supplementary figures and images for: Measuring nurses’ on-shift physical activity and sedentary time by accelerometry or heart rate monitoring: a descriptive case study illustrating the importance of context
Source: J Act Sedentary Sleep Behav. 2023 Dec 3;2:27. doi: 10.1186/s44167-023-00036-2 (PMC11960232; doi:10.1186/s44167-023-00036-2)

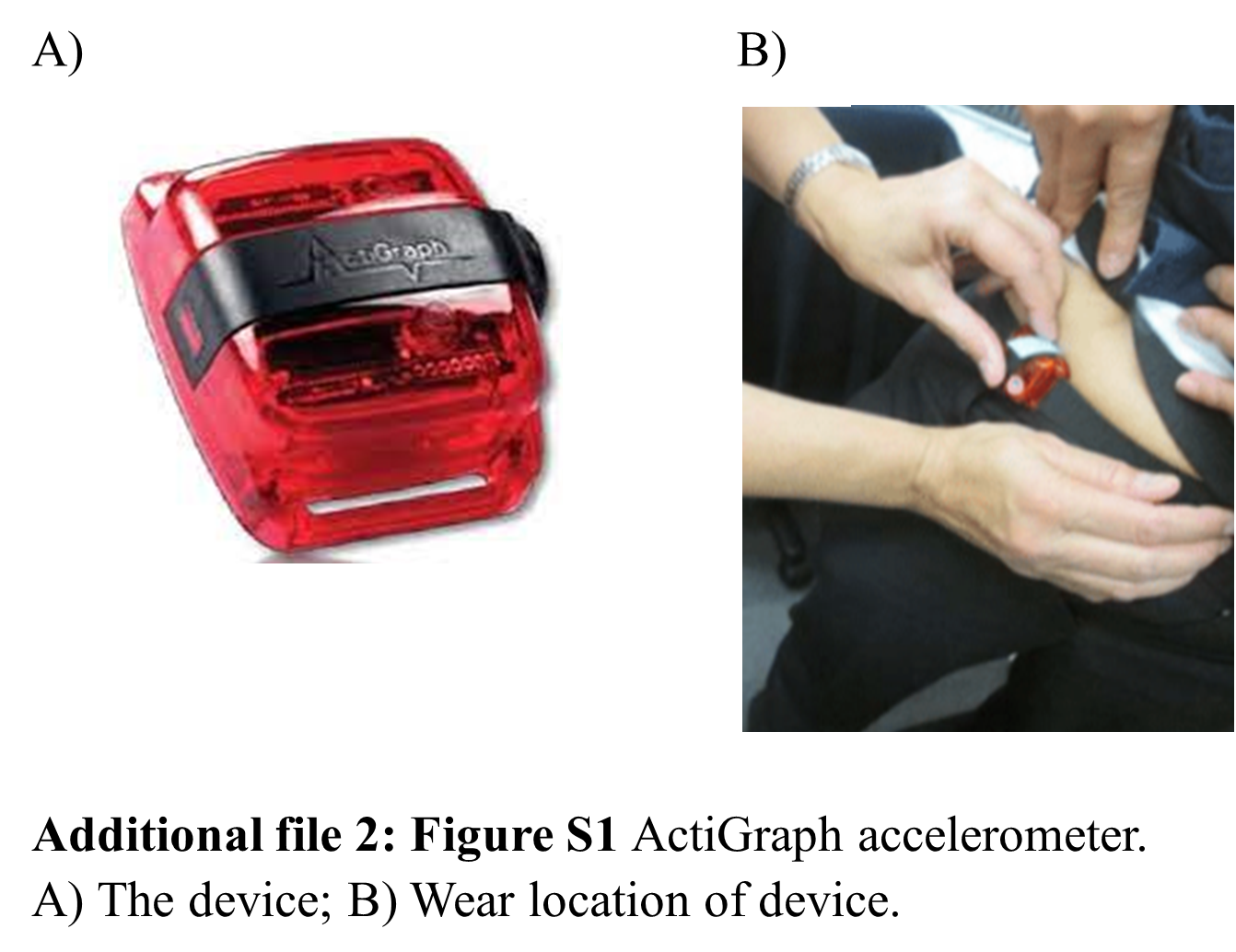

Supplement: Supplementary file 2 — Additional file 2: Figure S1. ActiGraph accelerometer. [file 44167_2023_36_MOESM2_ESM.tif]

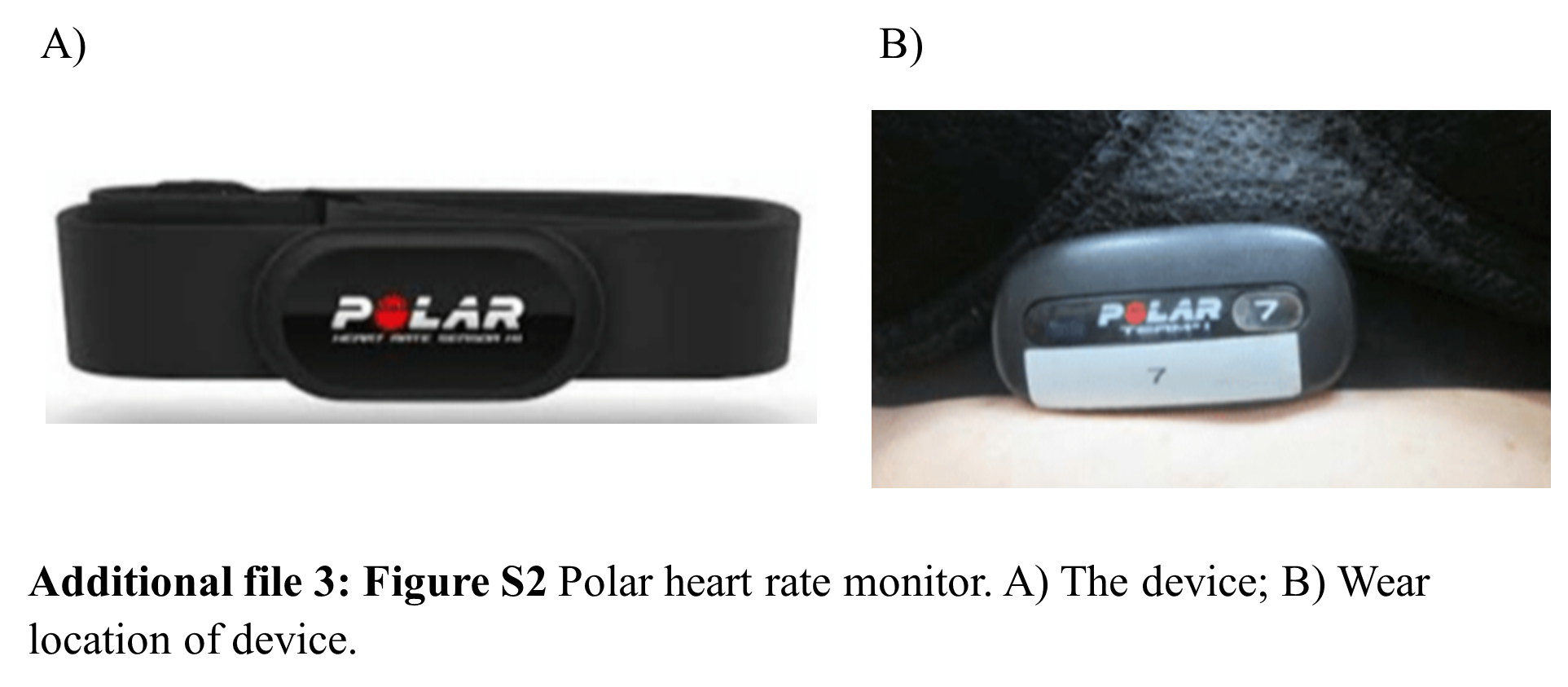

Supplement: Supplementary file 3 — Additional file 3. Figure S2. Polar heart rate monitor. [file 44167_2023_36_MOESM3_ESM.tif]
