# Supplementary material for: Measuring nurses’ on-shift physical activity and sedentary time by accelerometry or heart rate monitoring: a descriptive case study illustrating the importance of context
Source: J Act Sedentary Sleep Behav. 2023 Dec 3;2:27. doi: 10.1186/s44167-023-00036-2 (PMC11960232; doi:10.1186/s44167-023-00036-2)
Supplement: Supplementary file 4 — Additional file 4: Table S1. Physical activity intensities as a percentage of maximal heart rate. Table S2. Average percentage of time (%) spent in different activity levels from accelerometry and heart rate data. [file 44167_2023_36_MOESM4_ESM.docx]

**Additional file 4: Table S1.** Physical activity intensities as a percentage of maximal heart rate.

| **Intensity (37)** | **Definition** | **Maximal Heart Rate (36) (%)** | **Example** |
| --- | --- | --- | --- |
| SED  (≤1.5 METs) | Activities involving sitting or lying that require little movement and a low energy expenditure | <40 | Sitting whist completing paperwork |
| LPA  (1.6-2.9 METs) | Activity that does not cause a noticeable change in breathing rate across a sustained period | 40-50 | Standing or slow walking |
| MPA  (3-5.9 METs) | Activity that can be conducted whilst maintaining a conversation for a prolonged period | 51-70 | Fast walking or carrying light loads |
| VPA  (≥ 6 METs) | Activities where a conversation cannot be maintained | >70 | Fast running and heavy lifting |

SED, sedentary time; METs, metabolic equivalents; LPA, light-intensity physical activity; MPA, moderate-intensity physical activity; VPA, vigorous-intensity physical activity.

**Table S2** Average percentage of time (%) spent in different activity levels from accelerometry and heart rate data.

|  |  | **SED** | **LPA** | **MPA** | **VPA** |
| --- | --- | --- | --- | --- | --- |
| **All Shifts** | *Accelerometry* | 73 | 22 | 5 | 0.1 |
|  | *Heart Rate* | 13 | 35 | 52 | 0.2 |
| **Early Shifts** | *Accelerometry* | 62 | 31 | 7 | 0.1 |
|  | *Heart Rate* | 8 | 41 | 51 | 0.3 |
| **Late Shifts** | *Accelerometry* | 67 | 27 | 6 | 0.1 |
|  | *Heart Rate* | 18 | 27 | 55 | 0.2 |
| **Medical Ward** | *Accelerometry* | 75 | 20 | 4 | 0.1 |
|  | *Heart Rate* | 14 | 26 | 60 | 0.2 |
| **Surgical Ward** | *Accelerometry* | 58 | 35 | 7 | 0.1 |
|  | *Heart Rate* | 11 | 47 | 42 | 0.2 |

SED, sedentary time; LPA, light-intensity physical activity; MPA, moderate-intensity physical activity; VPA, vigorous-intensity physical activity.
